# Therapeutic Effects of Ten Commonly Used Chinese Herbs and Their Bioactive Compounds on Cancers

**DOI:** 10.1155/2019/6057837

**Published:** 2019-09-15

**Authors:** Wei Liu, Binbin Yang, Lu Yang, Jasmine Kaur, Calvin Jessop, Rushdi Fadhil, David Good, Guoying Ni, Xiaosong Liu, Tamim Mosaiab, Zhengjun Yi, Ming Q. Wei

**Affiliations:** ^1^Department of Laboratory Medicine, Key Laboratory of Clinical Laboratory Diagnostics in Universities of Shandong, Weifang Medical University, 261053 Weifang, Shandong, China; ^2^School of Medical Science & Menzies Health Institute Queensland, Griffith University, Brisbane, Australia; ^3^School of Allied Health, Australian Catholic University, Brisbane, Australia; ^4^Cancer Research Institute, Guangdong Pharmaceutical University, Guangzhou, China; ^5^Inflammation and Healing Research Cluster, Sunshine Coast University, Sunshine Coast, Australia; ^6^Molecular Diagnosis and Target Therapy Laboratory, Guangzhou, China

## Abstract

Effective cancer therapy is one of the biggest global challenges. Conventional cancer therapies have been at the forefront of combating cancers, but more evidence showed considerable side effects, limiting their use. There are various new therapies in development, but combined approaches for treating cancer are much expected. Natural herbs had been traditionally in use for cancer therapy in most parts of the world. In this review, we have examined ten commonly used Chinese herbs that have, for centuries, shown effectiveness in treating cancers. They demonstrated the abilities to promote the apoptosis of cancer cells, inhibit their metastasis, activate the patient's anticancer immunity, and synergistically increase the efficacy of conventional chemotherapy and radiation therapy when used in combination. Clinical experiences had proved that these herbs and their bioactive compounds were effective against a plethora of cancers through a variety of mechanisms, effectively improving patients' quality of life without significant side effects. These advantages indicate that there are huge potentials in the development of Chinese herbs into cancer medicine as part of a promising, holistic cancer treatment modality.

## 1. Introduction

Cancer is the leading cause of death worldwide, with an estimated 9.6 million people dying of cancer in 2018 [1]. It is groups of uncontrolled, cell proliferative, and chronic diseases, which pose an immediate threat to the life of patients with huge healthcare expenses [2]. Surgery, radiotherapy, and chemotherapy are now the most mainstreams of cancer therapy coupled with the emerging targeted and cancer immunotherapies [3]. To a certain extent, the growth and spread of cancer were controlled and the survival time of patients was prolonged by these methods, but the overall efficacy and many of the associated side effects were intolerable. In some cases, these treatments are effective, but the therapeutic effects did not last long, or cancer cells gained resistance to chemical drugs, limiting their further use. Therefore, it is necessary to develop a more ideal drug therapeutics or adjuvant treatment strategy for cancer.

Chinese herbs have demonstrated efficacy in the treatment and prevention of cancer through thousands of years of practice [4]. As a natural medicine, it has the advantages of low adverse reaction and low toxicity. There are over 2000 herbs registered in the Chinese Herbal register, which had been used of the treatment of a range of illnesses; about 400 have varying degrees of therapeutic effects on cancers, such as those of the lung, liver, brain, stomach, prostate, and breast [5]. The mechanisms may relate to the inhibition of cancer growth, reduction of metastasis and invasion, promotion of cancer cell apoptosis, and enhancement of immunity. Chinese herbal medicine is increasingly used as an alternative to mainstream cancer treatments [6]. Combination therapy with the use of herbs had produced more effective and lasting therapeutic effects. Furthermore, herbs can sensitize the body to radiotherapy and chemotherapy, increase the protection of normal tissues, and prevent tumor metastasis and recurrence. Chinese herbal medicine can not only inhibit local lesions but also regulate the whole body to eliminate cancers, without damage to normal cells [6]. In addition, Chinese herbal medicine can reduce the side effects of radiotherapy and chemotherapy, alleviate insomnia, depression, and fatigue, and significantly improve the quality of life. Therefore, herbs have unique advantages in improving the quality of life and extending the survival period of patients [6, 7].

The purpose of this article is to review the anticancer effects of ten commonly used Chinese herbs and their bioactive compounds for their abilities in enhancing immunity and killing cancer cells, thus providing rationales for the development of novel, strategic treatments for cancers.

## 2. Ten Commonly Used Herbs

There are numerous herbs and their many remedies/recipes, which have been used for cancer therapy through the past 2000 years in China and South and East Asia [8]. The mechanisms and effects are vastly different. In this review, we searched the top ten Chinese herbs with the highest number of results related to cancer on China National Knowledge Infrastructure (CNKI) and were presented here. Each herb has good anticancer properties and is widely used as cancer therapeutics.

### 2.1. *Oldenlandia diffusa* (OD, Bai Hua She She Cao in Chinese)

**Figure 1:**
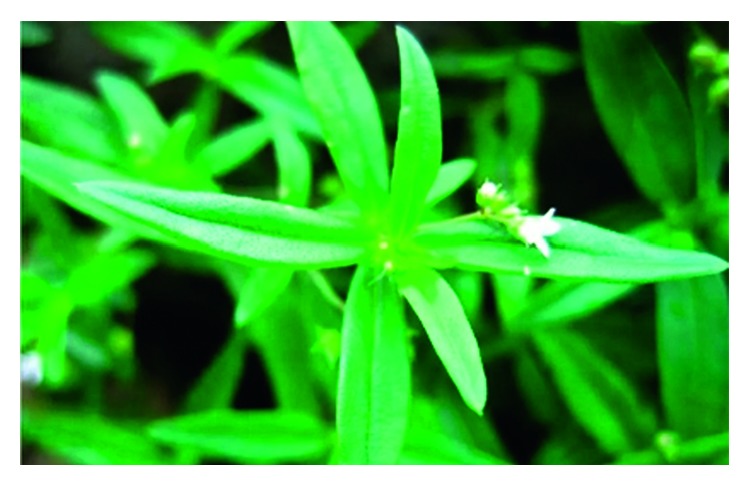
Photo of an *Oldenlandia diffusa* plant, with leaves and flowers.

OD as an annual herb has a wide range of pharmacological effects, including antioxidant, anticancer and immune regulation (Figure 1). It is one of the most well-known Chinese herbs for its anticancer effect. Chemical analysis has revealed that bioactive compounds including vanillic acid, 2-hydroxy-3-methyl-anthraquinone, 2-hydroxy-7-methyl-3methoxyanthraquinone, and 1-formaldehyde-4-hydroxyl anthraquinone are the main anticancer components of OD [7]. Vanoxalic acid and 2-hydroxy-3-methyl-anthraquinone are good tyrosinase inhibitors which have been used clinically to fight cancer. For clinical use, the ethanol-extracted OD had better efficacy.

OD and its active components can effectively induce apoptosis of prostate cancer, colorectal cancer, cervical cancer, gastric cancer, and other tumor cells [9]. It can reduce the proliferation and metastasis of liver cancer cells by inhibiting the expression of chemokine receptors such as CXCR1, CXCR2, and CXCR4 and induce apoptosis through caspase 3 pathway [10]. Chung et al. [11] found that OD inhibits the expression of MMP-9 to inhibit the invasion of breast cancer cells and regulates the expression level of apoptosis-related proteins to induce apoptosis of cancer cells. In addition, the water extract of OD leads to the apoptosis of lung cancer cells by inhibiting the MAPK pathway [5]. The expressions of apoptosis-related proteins Fas, caspase 3, and caspase 7 were significantly increased, and the expression of Fas L protein was inhibited in the kidney cancer model mice with the treatment of OD [12]. It can also induce apoptosis and inhibit the proliferation of bladder cancer T24 cells through the JAK2/STAT3 pathway [13]. In vivo experiments showed that total flavonoids of OD could significantly inhibit the tumor growth of cervical cancer in mice. The mechanism may be related to the increase of serum TNF-*α*, IFN-*γ*, and IL-2 levels [14]. Furthermore, the clinical application has proved that the herb can also achieve the effect of cancer control by enhancing body immunity [15].

As an anticancer drug, OD has a long history of clinical use with a good inhibitory effect on a variety of cancers, but it is often used in combination with other drugs to enhance its anticancer effects, most commonly in combination with *Scutellaria barbata*. It can be the best herb for antitumor therapy.

### 2.2. *Curcuma longa* (CL, Jiang Huang in Chinese)

**Figure 2:**
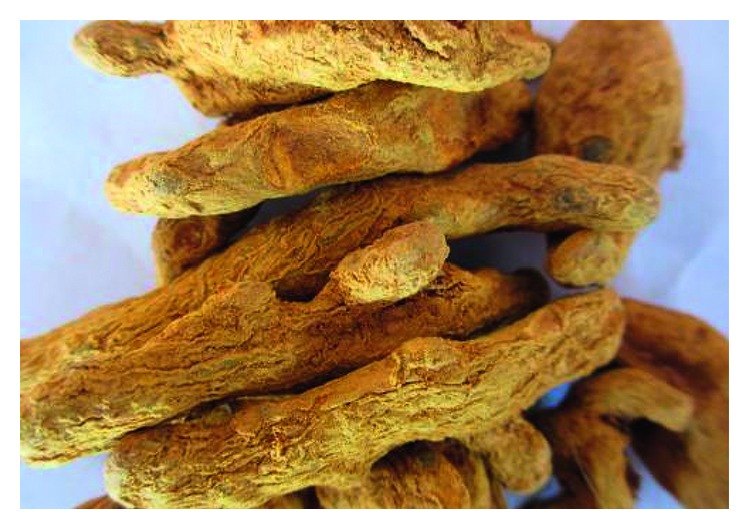
Photo of dry root *Curcuma longa*.

CL, known as turmeric, is a kind of perennial herb (Figure 2). Its main active ingredient is curcumin which has been used as a spice and pigment. Curcumin is a kind of diketone compound that has few side effects in humans. As a therapeutic agent, it is also used for the treatment of cancers and chronic diseases such as cardiovascular disease and diabetes. Turmeric is often taken as a single decoction.

Curcumin can inhibit the proliferation and migration of cervical cancer cells and cause the apoptosis of metastatic cells without affecting the survival of normal cervical epithelial cells [16]. Curcumin can also reverse TGF-induced epithelial-mesenchymal transformation (EMT) in hepatocellular carcinoma by downregulating Snail expression [17]. It has been reported that curcumin can inhibit the proliferation and migration of glioma cells and promote their apoptosis and its mechanism is closely related to the inhibition of the expression of oncogenic protein NEDD4 [18]. Similarly, curcumin has been found to affect the proliferation, invasion, and apoptosis of pancreatic cancer cells by inhibiting NEDD4 [19]. Curcumin may inhibit the expression of Cyclin B1 and downregulate the apoptosis-related protein Bcl-xl resulting in inhibition of the proliferation of thyroid cancer and promoting apoptosis [20]. Besides, curcumin significantly reduces the activity of oral squamous cell carcinoma cells by inhibiting the PI3K/AKT/mTOR signaling pathway [21]. In vivo experiment showed that gavage of curcumin to cervical cancer mice can effectively reduce the level of tumor marker molecule and nitric oxide, indicating the inhibition of cancer growth [22].

Turmeric is often used as a single drug to suppress the activity of different types of cancer, especially cancer of the digestive system from multiple perspectives. In recent years, curcumin has been proved to be an excellent natural compound against cancer, especially in the digestive system. So far, there are no reports that curcumin is toxic to animals or humans. These unique advantages of turmeric make it an excellent choice for the treatment and prevention of cancer.

### 2.3. *Astragalus membranaceus* (AM, Huang Qi in Chinese)

**Figure 3:**
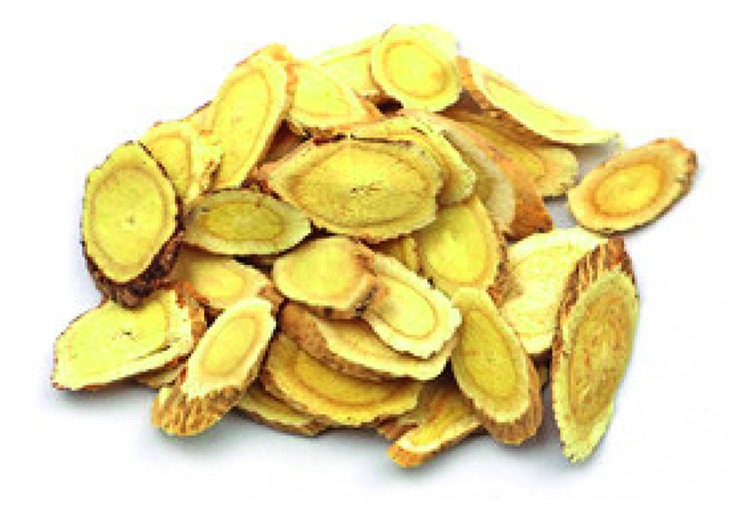
Photo of dry root *Astragalus membranaceus* slices.

AM is a very common Chinese medicine in China, belonging to the leguminous plant (Figure 3). Its main medicinal ingredients are astragalus polysaccharide and astragaloside, which have good immune and antiviral functions [23]. Astragalus polysaccharide is the most active ingredient in astragalus, and the higher concentration of ethanol extract has stronger activity. Astragalus injection is the most commonly used method of AM. It is also one of the main components of Buzhong Yiqi decoction, which is a traditional Chinese medicine prescription with good effect on cancers.

Studies have found that astragaloside can downregulate the expression of immune checkpoint proteins PD-1 and PD-L1 and inhibit the expression levels of migration-related proteins MMP-2 and MMP-9, which may be related to the inhibition of invasion and migration of cervical cancer cells [24, 25]. AM can also curb the expression of MMP-2 and MMP-9 proteins and inhibit the phosphorylation of ERK to control the proliferation and metastasis of ovarian cancer. AM can regulate the apoptosis of cancer cells by upregulating the apoptosis-related proteins caspase 3 and caspase 9 and increasing the proportion of Bax/Bcl-2 [26]. Zhou et al. [27] found that AM inhibits the proliferation of breast cancer cells through the PI3K/AKT/mTOR pathway and induce apoptosis of non-small cell lung cancer [28] and leukemia [29]. AM can also induce the polarization of macrophages into M1-type and activate the anticancer ability of macrophages [30]. Moreover, AM combined with other anticancer drugs such as cisplatin can significantly increase the inhibitory effect on cancer cells [31].

In recent years, AM has attracted much attention because of its good anticancer activity. AM has an obvious inhibitory effect on cervical cancer, breast cancer, lung cancer, and leukemia. AM can not only be taken as a single medicine but also used to synergistically enhance the efficacy of cisplatin. Therefore, it can be used as one of the research directions of anticancer herbs in the future.

### 2.4. *Panax ginseng* (PG, Ren Shen in Chinese)

**Figure 4:**
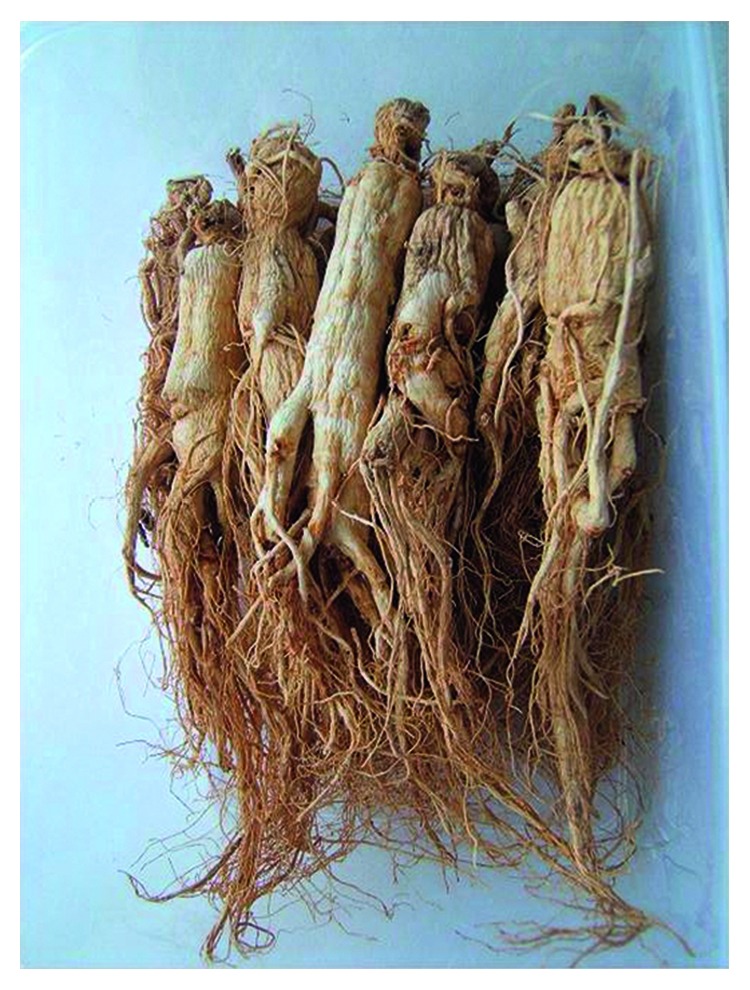
Photo of dry root *Panax ginseng*.

PG is the dry rhizome of the Araliaceous plant ginseng, which has attracted much attention due to its multiple pharmacological functions such as cardiovascular protection, antitumor, and antiaging [32] (Figure 4). Ginsenoside is the main active ingredient of ginseng, and some polysaccharides and amino acids also have some pharmacological functions. Ginsenosides are triterpenoids which have anticancer activities mainly include Rg1, Rg2, Rg3, Rh1, and Rh2. Ginseng is usually taken alone and is also the main ingredient of Lizhong decoction.

It is reported that Rh2 ginsenosides can promote endometrial cancer cells apoptosis, and its mechanism may be related to increased apoptosis markers cleaved poly ADP-ribose polymerase (PARP) and caspase 3 protein levels [33]. Ginsenoside Rg5 can regulate the miR125b/STARD13/NEU1 signaling pathway, which may be one of the mechanisms of ginseng in inhibiting invasion and migration of gastric cancer cells [34]. Meanwhile, ginsenoside Rg5 can also reduce Bcl-2 protein and increase the expression of Bax protein in gastric cancer cells, thereby reducing the activity of cancer cells [35]. When studying lung cancer cell A549, ginsenoside Rg3 induced apoptosis through upregulation of ROS formation and apoptosis protein caspase 3/9 and BAX [36]. Additionally, ginsenoside CK also has a good effect in inhibiting breast cancer invasion and metastasis and its mechanism is related to the downregulation of MMP-2 and MMP-9 expression [37]. Recently, it has been found that PG and astragalus water extract can regulate the polarization of macrophages toward M1-type and synergistically enhance the anticancer effect of cis-diamine dichloroplatin (DDP) [38]. In vivo experiments found that ginsenoside Rh2 can regulate the immune function of mice and significantly improve the killing effect of CD8+ T cells and NK cells on cancer cells [39].

As a star herb, PG has been widely used in Asia especially East Asia for thousands of years. In addition to its excellent anticancer properties, ginseng's immunomodulatory properties make it a good choice for anticancer herbs.

### 2.5. *Ganoderma lucidum* (GL, Ling Zhi in Chinese)

**Figure 5:**
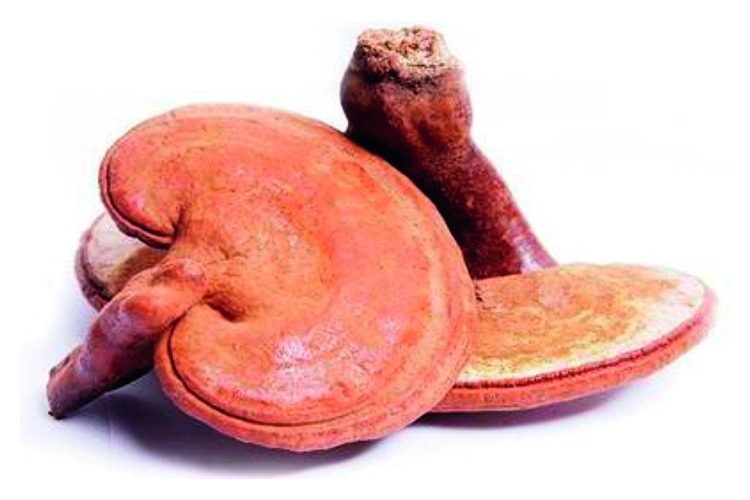
Photo of *Ganoderma lucidum*.

GL is a kind of fungus with both food and medicine (Figure 5). Besides its good health care function, GL can also manage diabetes and improve cardiovascular and anti-aging pharmacological effects. The active components of GL include polysaccharides, triterpenoids, proteins, amino acids, sterols, and alkaloids [40]. The polysaccharide is composed of three single sugar chains, with a spiral three-dimensional configuration of dextran; the three-dimensional configuration is similar to deoxyribonucleic acid DNA and RNA and is the most effective component of GL. GL is generally taken as a single drug, as a tonic drink, or food to take.

It has been reported that Ganoderma triterpenoids can inhibit the proliferation of lung cancer cells and the molecular mechanism is related to the regulation of cell cycle and the increase of Bax/Bcl ratio [41]. Triterpenoids can also downregulate MMPs to inhibit metastasis of prostate cancer cells and reduce cancer activity [42]. In breast cancer, GL extract reduces the number of breast cancer stem cells (BCSCs) by downregulating the STAT3 pathway to inhibit the invasion ability of cancer cells [43]. Furthermore, it has been reported that GL extracted with ethanol can enhance the protective autophagy of cells and inhibit the expression of EGFR and the PI3K/AKT/mTOR signaling pathway in chronic myelogenous leukemia cells [44]. Besides, GL polysaccharides can assist the anticancer effect of the chemotherapy drug paclitaxel (PTX) and inhibit the cancer metabolic process in the cancer microenvironment [45]. In addition to the direct killing of cancers, GL can also be used in immunotherapy against cancers. Studies have shown that GL polysaccharide-gold nanocomposite can activate dendritic cells and promote the proliferation of T cells and has a strong inhibitory effect on the proliferation and metastasis of lung cancer cells [46].

As a traditional Chinese medicine with both food and medicine, GL can be consumed by cancer patients in their daily diet. The effective ingredients in GL are very effective in the treatment of lung cancer, prostate cancer, breast cancer, and so on. GL can be used as an herbal treatment for cancer that is more easily accepted by patients.

### 2.6. *Angelica sinensis* (AS, Dang Gui in Chinese)

**Figure 6:**
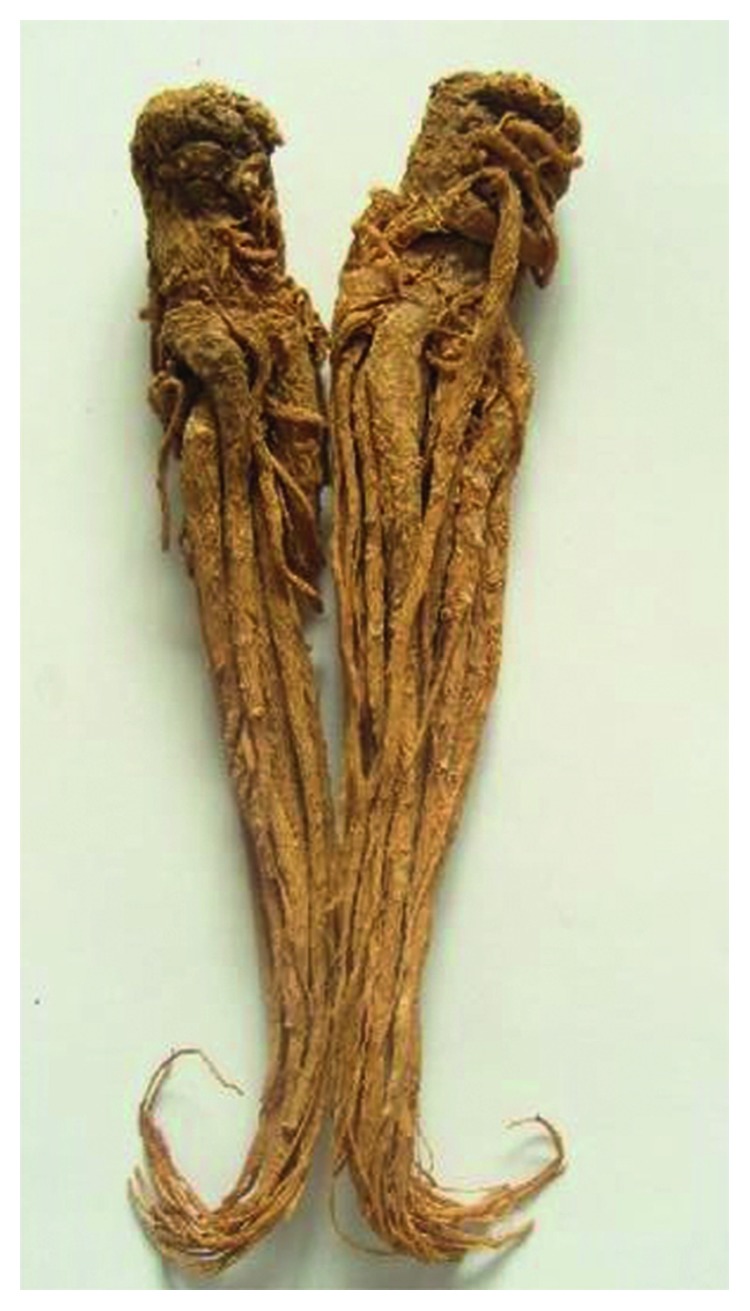
Photo of dry root *Angelica sinensis*.

AS belongs to perennial herbaceous plant, has extensive pharmacological action, has good cardiovascular, analgesic, and anti-inflammatory effects (Figure 6). It has many components, among which volatile oil, organic acid, and polysaccharides are the main [47]. The volatile oil is mainly composed of Z-ligustilide, which has anticancer and immunity-enhancement effect. AS is commonly used as a single food. Danggui buxue decoction is often used for the treatment of tumor-related anemia, which consists of AS and AM.

In studies on bladder cancer, it is found that N-butylidenephthalide extracted from Angelica inhibits the activity of cancer cells from multiple aspects, including upregulating caspase 3/9 to induce apoptosis of cancer cells, upregulating E-cadherin, and downregulating N-cadherin to inhibit cell migration. In combination with cisplatin, it can increase the sensitivity of cancer cells [48]. Angelica acetone extract significantly reduced the expression of hypoxia-inducible factor 1-alpha (HIF-1-*α*) and vascular endothelial growth factor (VEGF) in bladder cancer cells, effectively reducing the formation of cancer microenvironment. Meanwhile, the PI3K/AKT/mTOR signaling pathway of cancer cells is also inhibited [49]. Angelica polysaccharides induced breast cancer cells to express the cAMP-responsive element-binding protein (CREB), upregulated caspase 3/9, and cleaved PARP and led to apoptosis of cancer cells [50]. Studies have shown that high concentration of AS can inhibit the metastasis of lung cancer cells and the possible mechanism is not only to reduce the expression of MMP2, MMP-9, TGF-1, and metalloproteinase tissue inhibitor TIMP-1 but to increase the expression of TIMP-2 [51]. In addition, Angelica polysaccharide downregulates cyclins and Bcl-2, upregulates Bax, cleaves caspase 3 and E-cadherin, inhibits the TGF-signaling pathway and the growth activity of glioma cells in vivo and in vitro [52].

AS is often used in traditional Chinese medicine for cancer treatment and is an excellent choice for gynecological cancers such as breast cancer.

### 2.7. *Panax notoginseng* (PN, San Qi in Chinese)

**Figure 7:**
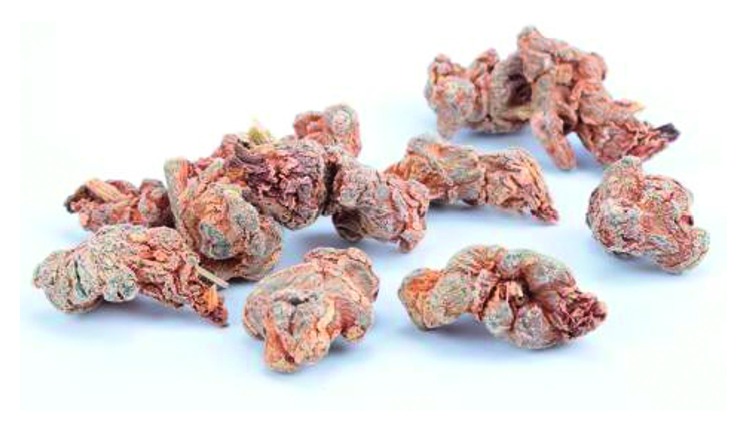
Photo of dry root *Panax notoginseng*.

PN is the dry root tuber of *Panax notoginseng*, which has strong pharmacological functions of enhancing immunity, antioxidation, antitumor, and antiaging (Figure 7). The main active components are *Panax notoginseng* saponins (PNS), in which ginsenoside Rb1, Rg1, Re, Rd, and PNS R1 play the main functions [53, 54]. In recent years, more and more studies have confirmed the efficacy of PN in the treatment of cancer, including the improvement of immunity and the destruction of the function of cancer cells. Powder made from PN is commonly used in traditional Chinese medicine.

It has been reported that *Panax notoginseng* ethanol extract (PNEE) can inhibit the invasion and metastasis of colorectal cancer cell line (HCT-116) by reducing MMP-9 and increasing the expression of E-cadherin, thus maintaining the integrity of the intercellular matrix. Also, PNEE reduces the protein levels of integrin-1 in HCT-116 and E-selectin and intercellular adhesion molecule-1 (ICAM-1) in endothelial cells EA.hy926, which reduces the adhesion ability of cancer cells [55]. PNS significantly inhibits the metastasis of breast cancer cells, and its function is to upregulate the expression of E-cadherin and cancer suppressor genes Brms1, Mtss1, and Timp2 and downregulate the expression of MMP3, MMP-9, and vimentin [56]. The growth of Lewis lung cancer cell line (LLC) treated with PNS was significantly reduced, and the expression level of Met/miR-222 axis was significantly reduced. And the expressions of cancer suppressor p27 and PTEN, which are the target genes of miR-222, were increased both in vivo and in vitro [57]. Furthermore, PNS can increase the cytotoxicity of cisplatin by enhancing the gap junction intercellular communication [58]. PN could regulate the polarization of macrophage M1, which can induce apoptosis of lung cancer cells by upregulating the apoptotic protein caspase 3/9, to reduce the volume of solid cancers [59].

*Panax notoginseng* can inhibit the activity of cancer cells through various mechanisms, enhance the immune cell function of cancer patients, and increase the efficacy of chemotherapy drugs. It has good effects on colorectal cancer, breast cancer, and lung cancer, and so on. It can help patients fight cancer in many aspects.

### 2.8. *Scutellaria barbata* D. Don (SB, Ban Zhi Lian in Chinese)

**Figure 8:**
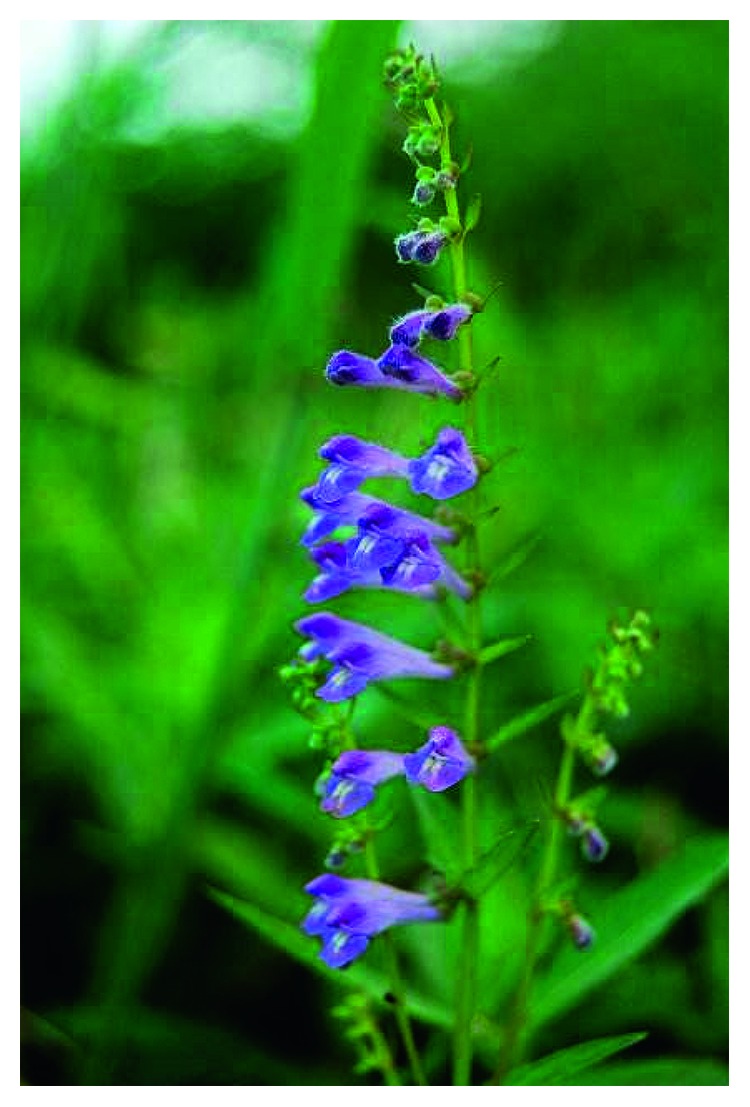
Photo of a *Scutellaria barbata* D. Don plant, with leaves and flowers.

SB is the whole herb of *Scutellaria barbata*, a perennial herb in the family Lamiaceae, which shows good efficacy in antitumor, antivirus, and antioxidation properties (Figure 8). The main active components of the herb are flavonoids, diterpenoids, and polysaccharides [60]. Scutellarin is the main ingredient in 10% of flavonoids. In traditional Chinese medicine theory, SB and OD are both heat-clearing and detoxifying drugs, so they are generally used in combination.

Studies on ovarian cancer cells showed that SB extracts downregulated Bcl-2 protein and increased caspase 3/9 protein to induce apoptosis. The migration ability of cancer cells was also inhibited, which may be closely related to the decreased expression of MMP-2/9 [61]. It inhibits the expression of HIF-1 and VEGF to inhibit the formation of cancer microenvironment and has been shown in vivo to inhibit the development of lung cancer and reduce capillary density [62]. Besides, polysaccharides of SB can increase the levels of Bax, Bak protein, and E-cadherin, decrease the levels of Bcl-2 protein, N-cadherin, and vimentin, inhibit the proliferation and EMT of colon cancer cells, and promote their apoptosis [63]. Studies have shown that the combined application of SB and OD can promote the apoptosis of bladder cancer cells by downregulating the expression of miR-155 and its regulated Akt signaling pathway. Meanwhile, the expression of McL-1 and Bcl-2 was also inhibited, and the expression of caspase 3 was upregulated [64]. In vivo experiments on mice showed that the ethanol extract of *Scutellaria barbata* (EESB) significantly reduced the expression of the Ki-67 protein, a cell proliferation marker of colorectal cancer. Also, the Wnt/*β*-catenin signaling pathway, proto-oncogene c-myc, and antiapoptotic protein survivin are all inhibited [65]. Moreover, EESB inhibits il-6-mediated STAT3 activation in colorectal cancer cells and downregulates the expression of cyclin D1 and CDK4 [66]. An experiment on C26 tumor-bearing mice showed that SB polysaccharide can inhibit tumor growth by activating caspase 3/9 [67].

The combination of *Scutellaria barbata* and *Oldenlandia diffusa* can enhance the anticancer effect of each other, which is the key point of anticancer herbs. It mainly treats digestive tract cancers, gynecological cancers, lung cancer, bladder cancer, and other cancers.

### 2.9. Licorice (Gan Cao in Chinese)

**Figure 9:**
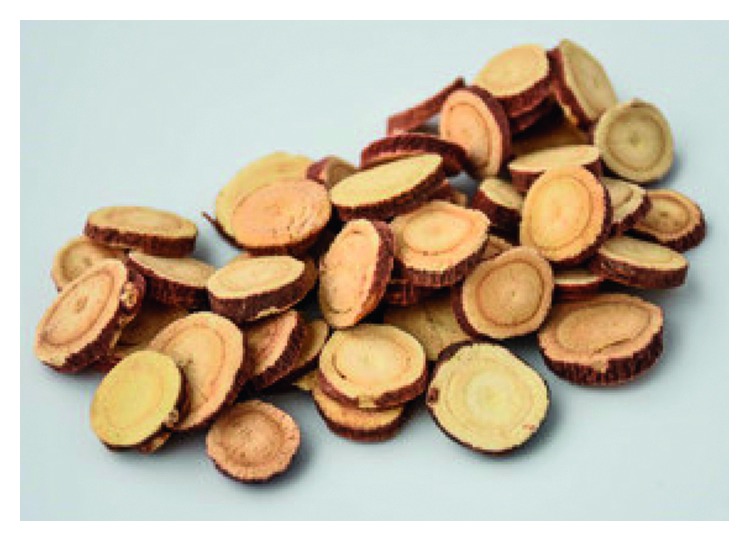
Photo of dry root licorice slices.

Licorice is the dry root and rhizome of the leguminous plant *Glycyrrhiza uralensis* (Figure 9). The main active ingredient of licorice is glycyrrhizic acid (or glycyrrhizin), which is a saponin structurally. Licorice has a clinical application history of more than 2,000 years and has a variety of pharmacological effects such as antibacterial, antiviral, anti-inflammatory and antitumor [68]. Licorice tablet is the most commonly used form.

It is reported that mitochondrial ROS production in breast cancer cells treated with glycyrrhizin was significantly increased. Meanwhile, the expressions of the apoptosis-inducing factor (AIF) and the autophagy protein LC-3 were upregulated, indicating that glycyrrhizic acid simultaneously regulates the apoptosis and autophagy process of cancer cells and mediates their death [69]. Glycyrrhizin can induce apoptosis of oral cancer cells by increasing the ratio of Bax/Bcl-2 and upregulating the cleavage of caspase 3/9 and PARP [70]. In addition, 18*β*-glycyrrhizic acid can inhibit the metastasis and invasion of gastric cancer cells, which may be related to the decreased expression of MMP-2/9 and vimentin, and upregulate the expression of E-cadherin. At the same time, this study found that 18 glycyrrhetinic acids could inhibit EMT of cancer cells by inhibiting ROS/pkc-/ERK signaling pathway [71]. Moreover, the protein extract of licorice also inhibited the activity of gastrointestinal cancer cells to some extent but had no significant effect on noncancer cells [72]. In in vivo experiments on mice, it was found that glycyrrhizin inhibited cancer growth, resulted in weight gain of immune organs and activation of peripheral blood T lymphocytes, and some cytokines, such as IL-2, IL-6, and IL-7 were increased [73]. Besides, it has been reported that licorice extract reverses cisplatin resistance in breast cancer cells by inhibiting cytochrome P450 1B1 enzyme [74].

Licorice is commonly used for cancer therapy of the digestive system, breast cancer, lung cancer, and so on. And it has the advantages of low price and wide distribution.

### 2.10. Radix *Salvia miltiorrhiza* (SM, Dan Shen in Chinese)

**Figure 10:**
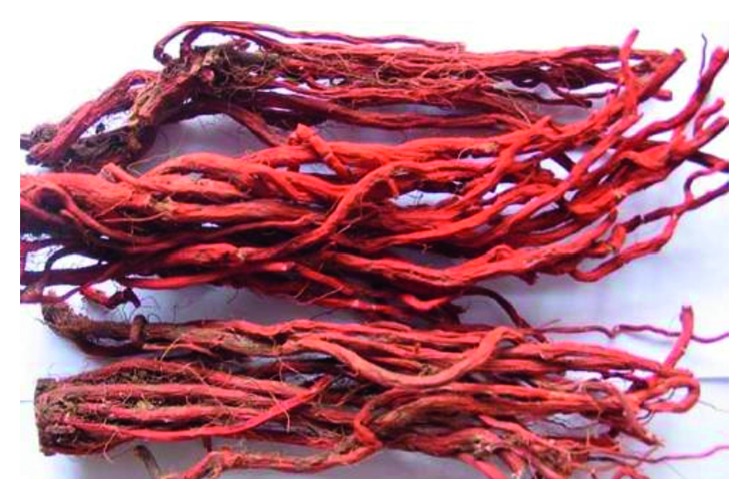
Photo of dry root *Salvia miltiorrhiza*.

SM is the dry root and rhizome of *Salvia miltiorrhiza* in Lamiaceae (Figure 10). The main active ingredients of SM include tanshinone, etc., which are fat-soluble phenanthraquinone compounds with good anticancer activity [75]. SM injection is the most commonly used form in traditional Chinese medicine. It is often used in combination with astragalus.

Studies have shown that ethanol-extracted tanshinone significantly inhibits the activity of gastric adenocarcinoma, prostate cancer, breast cancer, colorectal cancer, and lung adenocarcinoma, respectively [76]. In the study of breast cancer cell line MCF-7, it was found that SM extract reduced the ability of metastasis and invasion of cancer cells by inhibiting the expression of MMP-9 mediated by MAPK/ap-1 signal transduction pathway [77]. The dihydrotanshinone in SM can inhibit the proliferation of glioma and promote its apoptosis, possibly through the increased cytochrome c cytoplasmic level and the activation of caspase 3/9, thereby PARP is lysed [78]. In studies on leukemia cells, it was found that 15,16-dihydrotanshinone I increased the phosphorylation of JNK and the expression of Fas L, which may be the reason for the upregulation of Bad and Bax expressions as well as the activation of caspase 3/8/9, leading to the apoptosis of leukemia cells [79]. Salvia acetonitrile extract induces the production of ROS in prostate cancer cells, reduces the expression of cyclin, and increases the expression of apoptosis-related proteins [80]. Besides SM extract, the mixture of SM and astragalus can reduce the expression of TGF-protein and nuclear input protein imp7/8, and the expression of TGF-specific serine/threonine kinase receptor T*β*RI and T*β*RII is also inhibited [81].

SM and tanshinone have a wide range of anticancer effects to inhibit lung cancer, breast cancer, and cancer of the digestive system. It can also remove blood stasis in cancer patients and is also an excellent choice of anticancer herbs.

## 3. Discussion

Traditional Chinese medicine (TCM) is becoming more and more popular in cancer treatment as a treatment modality or as complementary and alternative medicine (CAM). Conventional chemotherapy and radiation therapy not only kill cancer cells but also cause many side effects to patients. There were also associations with chronic pain, fatigue, and depression. In China, at least a high percentage of cancer patients opt for traditional Chinese medicine to reduce side effects. The Chinese herbal medicine administration method has been continuously improved for thousands of years, and now, it is mainly based on powder, decoction, and injection.

Besides, topical medication is also a good choice, which maintains anticancer drugs in high concentration and long-lerm effect on cancer tissue. Our research found that Chinese herbal medicine can change the gene expression of cancer cells. Similar results have been achieved in animals. However, it is not enough to confirm the therapeutic effect of Chinese herbs on cancer. Further trials or clinical trials are needed to prove its efficacy. We speculate that these mechanisms may be related to the role of Chinese herbal medicine in the human body and hope to provide a theoretical basis for future clinical trials. In addition to the ten herbs mentioned above, other Chinese herbs and their preparations, such as *Radix isatidis*, *Pinellia ternata*, and *Taxus chinensis*, have also been recognized by the world in the treatment of cancer. These herbs are widely distributed and more readily available to cancer patients. And the price is much lower than common anticancer drugs, which is affordable to patients.

The usage method is important for the application of Chinese herbal medicine. The correct usage of Chinese herbs can double the effect of cancer therapy with half the effort, while excessive use may have adverse effects on the human body. It has been reported that OD causes acute kidney damage in the treatment of cervical cancer [82]. The reason is the renal toxicity caused by long-term use of OD in the common dose, which improved after a period of discontinuation. Some ingredients in traditional Chinese medicine can also cause drug-induced liver damage [83]. The hepatotoxicity of most traditional Chinese medicines is positively correlated with dose. Different processing methods also affect the level of drug toxicity. Reasonable compatibility and the scientific method should be adopted to reduce its toxicity as far as possible [84]. The decoction used in traditional Chinese medicine is diluted, and the toxicity of the medicine is reduced. Both traditional Chinese medicine tablets and injections have undergone long-term toxicity tests, and patients should follow the doctor's advice or instructions when using them. In short, the vast majority of Chinese herbal medicine, if used correctly, does not produce significant side effects.

## 4. Conclusions

This article introduces ten kinds of Chinese herbal medicines which can be used against cancers in different ways mainly by promoting the apoptosis of cancer cells, inhibiting the activity of cancer cells, and enhancing immunity. In the future, it may be possible to use this natural product with low side effects to fight cancer.
